# Extracorporeal membrane oxygenation in critically ill patients with active hematologic and non-hematologic malignancy: a literature review

**DOI:** 10.3389/fmed.2024.1394051

**Published:** 2024-10-22

**Authors:** Roberto Rabello Filho, Daniel Joelsons, Bruno de Arruda Bravim

**Affiliations:** Department of Critical Care Medicine, Hospital Israelita Albert Einstein, São Paulo, Brazil

**Keywords:** extracorporeal membrane oxygenation, hematology, oncology, malignancy, intensive care unit

## Abstract

Combined progress in oncology and critical care medicine has led to new aspirations and discussions in advanced life support modalities in the intensive care unit. Over the last decade, extracorporeal membrane oxygenation, previously considered unsuitable for oncologic patients, has become increasingly popular, with more diverse applications. Nevertheless, mortality remains high in critically ill cancer patients, and eligibility for extracorporeal membrane oxygenation can be extremely challenging. This scenario is even more difficult due to the uncertain prognosis regarding the underlying malignancy, the increased rate of infections related to intensive care unit admission, and the high risk of adverse events during extracorporeal membrane oxygenation support. With advances in technology and better management involving extracorporeal membrane oxygenation, new data on clinical outcomes can be found. Therefore, this review article evaluates the indicators for extracorporeal membrane oxygenation in different types of oncology patients and the possible subgroups that could benefit from it. Furthermore, we highlight the prognosis, the risk factors for complications during this support, and the importance of decision-making based on a multidisciplinary team in the extracorporeal membrane oxygenation indication.

## 1 Introduction

The continuous development in medical technology and chemotherapeutic agents has improved the prognosis of patients with solid and hematological malignancies (1, 2). In particular, the advances in hematopoietic cell transplantation (HCT) have expanded patient eligibility, with good disease-free outcomes (3, 4). Combined progress in oncology and critical care medicine has led to new aspirations and discussions about advanced life support modalities, such as extracorporeal membrane oxygenation (ECMO), previously considered unsuitable for these patients (5, 6).

Nevertheless, mortality remains high in critically ill oncology patients. It is mainly associated with some factors, such as emergency medical admissions, treatment-related reactions, organ dysfunction, and disease progression (7, 8). The main reason for intensive care unit (ICU) admission among patients with cancer is acute respiratory failure, and the mortality rate in patients requiring mechanical ventilation is above 70% (9).

For patients with severe acute respiratory failure in whom conventional mechanical ventilation fails, veno-venous extracorporeal membrane oxygenation (VV-ECMO) can be a rescue treatment option (10). Veno-venous ECMO provides extracorporeal gas exchange to maintain carbon dioxide removal and adequate oxygenation to avoid ventilator-induced lung injury (VILI) until the cause of severe respiratory failure has resolved (11). Furthermore, ECMO can also provide circulatory support. Veno-arterial ECMO (VA-ECMO) is indicated in cardiogenic shock to maintain a patient’s systemic perfusion as a bridge to myocardial tissue recovery, destination therapy with left ventricular assist device placement, or heart transplant (12).

Over the last decade, ECMO as a potentially lifesaving intervention has become increasingly popular, with more diverse applications. Technological advances in circuits and improvements in clinical management have resulted in better outcomes (13, 14). Recently, data from the registry of the Extracorporeal Life Support Organization (ELSO), which manages the record of operational ECMO centers globally, indicated a hospital discharge rate of 58% in adults and 69% in pediatric pulmonary support with ECMO (15). In particular, support in cases of severe acute respiratory distress syndrome (ARDS) has caused a rise in the use of ECMO in oncology patients (16).

Despite these advances, patient selection criteria for eligibility for ECMO is an ongoing controversy. This is especially true for cancer patients, given the uncertain prognosis regarding the underlying malignancy, the increased rate of infections related to ICU admission, and the high risk of severe ECMO-related adverse events (17). Consequently, ELSO includes immunosuppression as a relative risk factor for ECMO indication, and the arbitrary indication of ECMO resources can place an elevated financial strain on the healthcare system (11, 18). Therefore, given the complexity of this topic, this study aimed to review how ECMO can be indicated to support different types of oncology patients, evaluate the risks and prognosis of oncology patients who receive ECMO, and discuss the subgroups of oncology patients who could benefit from ECMO.

## 2 ECMO and cardio-respiratory support

With the development of better capacity to inhibit tumor growth, new treatment options for severe cancer patients are emerging. The increase in life expectancy also brings a greater risk of associated complications, and, among them, acute respiratory failure stands out. Possible indications of ECMO among these patients may have room for discussion. With advances in technology and better care involved in patients undergoing ECMO therapy, new data on clinical outcomes can be found. However, solid tumors and hematological malignancies differ in their pathogenesis, treatment, complications, and patient prognosis; therefore, they should be discussed separately.

### 2.1 Solid tumors

Gow et al. evaluated data from 72 adult oncologic patients who were considered for extracorporeal life support; of these, 47 had solid tumors, 21 had hematologic malignancies, and 4 underwent hematopoietic stem cell transplantation. Among these patients, 54 required ECMO for respiratory support, and the median duration of ECMO was 4.1 days. Overall, 44 patients (61%) died on ECMO, 23 patients (32%) survived hospital discharge, and 5 patients (7%) survived during ECMO assistance but died before discharge. The authors found that pulmonary support as a reason for ECMO, impaired lung function before ECMO, and the development of infection were the major risk factors for death in this population (19).

In 2015, Wu et al. conducted another retrospective study evaluating 14 cancer patients (solid malignancies in 13 patients and hematological malignancy in 1 patient) who received VV-ECMO for severe acute respiratory failure that developed within 3 months after anticancer therapies. The indication of VV-ECMO was a PaO_2_/FiO_2_ ratio of less than 70 mmHg under advanced mechanical ventilation. The authors found a median ECMO weaning rate of 50% (7 patients) and a hospital survival rate of 29% (4 patients). Survival was not observed among patients presenting with progressive or recurrent malignancy, severe neutropenia, or acute renal failure necessitating dialysis during the ICU stay (20).

In a more recent and larger cohort of cancer patients treated with VV-ECMO, Kochanek et al. analyzed retrospectively the clinical characteristics and outcomes of 297 cancer patients from 19 German and Austrian hospitals who underwent VV-ECMO between 2009 and 2019. In this population, 59 (54%) had a solid tumor, and 138 (47%) had a hematologic malignancy. The survival rate for the entire population was 26.8% at 60 days. Platelet count, elevated lactate levels, and disease status (progressive disease or newly diagnosed) were independent adverse prognostic factors for mortality. The authors also observed a high rate of severe bleeding episodes (38%), and severe hemorrhage episodes during ECMO were associated with increased ECMO-related mortality. The main clinical factors that were correlated with an increased risk of bleeding were thrombocytopenia and recent chemotherapy, justifying the negative prognostic impact of thrombocytopenia in the analyses. In addition, a propensity score-matched analysis demonstrated that the outcome of cancer patients receiving ECMO was not superior to patients treated only with conventional mechanical ventilation (16).

Another large study on this topic reported the outcomes of 203 immunocompromised patients with severe ARDS requiring VV-ECMO, including 101 patients with cancer. The overall outcome of cancer patients receiving VV-ECMO support as a rescue treatment for acute respiratory failure was also poor. The survival rate of cancer patients in this study was comparable to that in the study by Kochanek et al. (6-month overall survival of 30% versus a 60-day overall survival of 26.8%). Most deaths occurred within the first 30 days of ECMO, and after 60 days, the Kaplan–Meier survival curve reached a plateau (17).

The combined evaluation of these data highlights the insufficient knowledge to select patients who can benefit from VV-ECMO and the importance of a careful multidisciplinary approach for indicating ECMO support. Despite the high mortality demonstrated in this subgroup of the critically ill patient population, judicious use of ECMO support could be beneficial to a very small population of oncology patients. Factors, including organic dysfunctions, thrombocytopenia, neutropenia, and, mainly, oncological disease status are indicators of poor prognosis that should be accounted for in the decision to provide VV-ECMO in cancer patients. Considering the methodology of the studies evaluated, the oncologic patient prognosis on ECMO requires further investigation. Studies specifically evaluating the prognosis in solid tumor patients with high functional status are needed.

### 2.2 Hematological malignancies

Recent advances in chemotherapy and hematopoietic stem cell transplant (HSCT) have benefited patients with hematologic malignancies (HM) such as leukemia, lymphoma, and multiple myeloma (21). Despite this, up to 50% of hospitalized inpatients may require invasive mechanical ventilation (MV), and the need for MV is associated with a worse outcome in patients with HM admitted to the ICU (22).

However, current studies have shown that the ICU mortality rates of HM patients have declined significantly over the last two decades (23, 24). A large prospective, observational cohort study performed in 17 centers in France and Belgium evaluated 1,011 patients to assess the outcomes of HM patients admitted to the ICU. The authors demonstrated a mortality rate of 39.3%, significantly lower than in previous studies. Moreover, the subsequent disease-control rate of 6 months after discharge from the hospital was 80% (24). Although patients on ECMO were not specifically analyzed, this study revealed that this population might have demonstrated better survival rates in the ICU.

However, the complex nature of patients with HMs may increase the risk of complications in the ICU, especially under ECMO support (24, 25). The additional use of chemotherapy increases the incidence of cytopenias and extended periods of marrow aplasia, making the patients more susceptible to viral, bacterial, and fungal infections (26). Furthermore, prolonged use of ECMO is also associated with a greater risk of infection (25). Thus, the initiation and management of ECMO support in these patients must be even more careful.

Recently, studies involving a variety of adults with HMs were conducted to assess the effect of ECMO on clinical outcomes. In a study conducted in South Korea, Kang et al. performed a retrospective review of clinical outcomes in 15 patients with HMs under ECMO support after the failure of optimal conventional therapy. Among the patients with HMs, ten had leukemia, including eight with acute myelogenous leukemia and two with acute lymphoblastic leukemia. Their outcomes were compared to 33 immunocompetent patients with cardiorespiratory failure who also needed ECMO support. While the incidence of bleeding events in the HMs group did not differ from that in the immunocompetent group, the HM patients presented significantly higher mortality rates, partially attributed to infections and hyperbilirubinemia during ECMO (27).

These findings are supported by another retrospective cohort study of 14 adult patients with HMs, all of whom received ECMO support due to severe acute respiratory failure, in which the incidence of bleeding complications was not higher in patients with hematologic malignancies than in control patients. The population included a variety of aggressive HMs, including non-Hodgkin lymphoma, Hodgkin lymphoma, acute myeloid leukemia, and multiple myeloma. Eleven patients received VV-ECMO, and three patients received VA-ECMO. The results were better than in previous studies, showing that 50% of the patients survived the ICU and hospital stay. Moreover, the long-term survival was 100% after a 36-month follow-up after hospital discharge (28). This study was the first to demonstrate that ECMO can be feasible for selected patients with HMs, with favorable long-term outcomes.

The reasons for the improved results of the study by Wohlfarth et al. should be thoroughly discussed. First, the sample size was small, with highly selective patients. In addition, most patients in the Wohlfarth et al. study had lymphoma, and, in comparison to other hematological malignancies, acute leukemia presents a poor prognosis in the ICU (22). Second, the retrospective design of the study did not allow conclusions about the true efficacy of ECMO in HM patients. Third, the data reflected the experience of a single institution specifically dedicated to the treatment of cancer patients and ARDS, including ECMO support. This calls for systematic prospective studies in the future with a larger sample size.

#### 2.2.1 Hematopoietic stem cell transplantation

Pulmonary complications are considered a major source of morbidity and mortality following allogeneic hematopoietic stem cell transplantation (HSCT) (29). Acute respiratory distress syndrome can develop in about 16% of malignant and non-malignant hematologic patients undergoing allogeneic hematopoietic stem cell transplantation and about 3% of patients undergoing autologous hematopoietic stem cell transplantation in the first year of HSCT (30). These findings suggest that ECMO can be discussed with HSCT recipients with severe ARDS as a rescue therapy in refractory cases.

A retrospective multicenter study conducted in 2017 analyzed 37 HSCT recipients treated with VV-ECMO for ARDS to evaluate outcomes in this specific population of critically ill patients. ICU admission occurred at a median of 146 days after allogeneic HSCT, and the main reason for ARDS was pneumonia (81% of patients). The number of complications was high; 14 patients (38%) experienced bleeding events, of which 6 (16%) events were associated with fatal outcomes (31). Overall, 7 patients (19%) survived hospital discharge and were alive after an 18-month follow-up. However, 6 of 13 (46%) patients who started ECMO after 240 days of HSCT survived (32). These findings suggest that survival rates in post-HSCT patients do not support the use of ECMO for ARDS in this group. On the other hand, long-term allogeneic HSCT recipients may be potential candidates after careful selection.

A retrospective monocentric study conducted in 2018 assessed 25 cancer patients treated with ECMO for ARDS, 11 of whom had undergone HSCT. The main reason for ARDS was also pneumonia (72%), and all patients were under invasive MV at ECMO support. Overall, 17 (68%) of these patients died, and only 20% survived until hospital discharge (35%). All patients after recent allogeneic HSCT died. Furthermore, the number of severe bleeding events was once again high (33). Accordingly, in these retrospective studies described above, it appears that except for specific cases, HSCT recipients complicated by ARDS treated with ECMO have a high risk of complications and a high mortality rate, discouraging the routine use of ECMO in this population.

## 3 ECMO for surgery support

In cardiopulmonary surgeries, the need for hemodynamic support such as cardiac bypass is a common occurrence. Recent trends favor the use of ECMO over traditional extracorporeal circulation (ECC) for a variety of reasons. The predominant advantage of ECC lies in its capacity for a complete replacement of cardiopulmonary bypass (CPB), coupled with the potential for autotransfusion. On the other hand, VA-ECMO with peripheral cannulation offers several distinct benefits. These include the method of peripheral cannulation itself, the requirement for lower anticoagulation levels (which are further reduced in VV-ECMO), minimized cardioplegia, a circuit characterized by enhanced biocompatibility, and a diminished inflammatory response (31, 34, 35).

In cases of pulmonary tumors, surgery can serve as a crucial adjunct to the curative process of cancer treatment. Particularly in advanced neoplasms, with extensive disease or tumors located in unfavorable positions, the use of cardiac bypass during the intraoperative phase may be necessary. Surgery, in these instances, becomes an essential component of a comprehensive treatment strategy, addressing the complexities and challenges posed by such critical tumor locations. In a high-volume French center, over 30 years, 201 patients underwent surgeries for invasions into the superior vena cava, carina, thoracic aorta, left atrium, or pulmonary artery trunk. Of these, merely 13 cases necessitated the use of CPB, suggesting that the need for extracorporeal support was a rare event in such procedures (36).

Byrne et al. published their experience in highly complex surgeries for thoracic neoplasia. Over a decade, they performed 14 surgeries using extracorporeal circulation, a time when ECMO was not the preferred option and the use of ECC itself in such cases was controversial. Among the patients who underwent surgery for resection of locally advanced thoracic malignancies, eight cases were pre-planned with centrally located tumors, while six cases needed emergency ECC due to injury to the superior vena cava (two cases), inferior vena cava (two cases), or pulmonary artery (two cases). Remarkably, complete tumor resection was achieved in 12 of these patients, accounting for an 86% success rate. There was one intraoperative fatality caused by pulmonary embolism; notably, all other patients recovered successfully and were discharged from the hospital (37).

In a retrospective study conducted in Vienna, Lang et al. described their group’s experience with oncological surgeries, specifically employing VA-ECMO. The study involved a total of nine patients undergoing surgeries, comprising six cases of complex tracheobronchial resections and three cases of resections involving major thoracic vessels. The team successfully achieved total tumor resection in eight patients. There was a single perioperative death attributed to hepatic necrosis. Impressively, the study reported a 5-year survival rate of 76.7% (38).

In a meta-analysis conducted by Muralidaran et al., 20 articles were reviewed focusing on the use of CPB in primary lung cancer resections. The study encompassed a total of 72 patients, with 74% undergoing pneumonectomy. Additionally, 43% of the cases involved resection of the aorta, 25% involved the left atrium, and 11% involved the pulmonary artery. The overall 5-year survival rate was 37%. Notably, the results of the multivariate analysis revealed that the planned versus unplanned use of CPB was the only factor significantly associated with survival, showing an odds ratio of 0.28 with a 95% confidence interval (CI) of 0.09 to 0.90 (39).

## 4 Bridge for chemotherapy

The literature on this topic is scarce, with the majority being case reports and expert opinions. Meltzer et al. reported a case involving a 40-year-old patient with lymphoma encasing both the right and left ventricles. The patient experienced decompensation from heart failure before the induction of chemotherapy, which precluded the initiation of therapy. In this scenario, VA-ECMO was proposed as a bridge to treatment, enabling the commencement of chemotherapy. The patient subsequently showed tumor remission and successful decannulation from ECMO (40).

Allain et al. reported a case involving a 65-year-old man who experienced cardiogenic shock and required support with VA-ECMO. Following the stabilization of his condition, the patient was diagnosed with primary cardiac lymphoma. Subsequently, chemotherapy was administered, and the patient showed hemodynamic improvement, leading to the withdrawal of ECMO support and the recovery of cardiac function (41).

In another case report, Chung et al. described a 22-year-old woman who initially presented with symptoms of dyspnea and was diagnosed with pulmonary thromboembolism. Faced with hemodynamic instability, the patient required the implementation of VA-ECMO. Subsequently, the patient underwent a pulmonary embolectomy to treat her critical circulatory collapse. Despite the procedure, her clinical condition did not improve. Further histopathological examination revealed the presence of metastatic choriocarcinoma. Given her persistent hemodynamic instability, the medical team decided to initiate chemotherapy supported by ECMO. This intervention led to a positive treatment response, and after a 3-month hospital stay, she was successfully discharged (42).

In conclusion, the literature on using ECMO as a bridge to chemotherapy is quite limited, predominantly restricted to case reports. There is the potential for significant bias in this area, as it is likely that only cases with positive outcomes are reported. Instances where patients do not survive are less frequently published. The ELSO itself categorizes such patients as having a relative contraindication to ECMO. Therefore, with careful clinical judgment and discernment, a small subset of patients might benefit from ECMO to initiate chemotherapy.

## 5 The role of the multidisciplinary team

Given the rate of complications and the high risk of severe ECMO-related adverse events in oncologic patients, the routine use of ECMO in this population is discouraged. It is clear from the data demonstrated above that the population of oncology patients has high mortality when ECMO is required. For solid tumors, the long-term survival rates range from 26 to 32%. In patients with hematological malignancies, studies demonstrate high variability, with mortality rates ranging from 50 to 100%. In this specific subgroup, there are few studies available and a low number of patients are analyzed. Therefore, both the initiation and management of ECMO support must be careful in this population.

The high mortality rate in cancer patients treated with ECMO can be explained by the complications associated with the severity of the oncologic disease, in addition to the inability to manage the ECMO support. However, a team approach with intensive multidisciplinary discussions demonstrated a good experience with the possibility of achieving good outcomes with ECMO for cardio-respiratory support in an extremely severe population during the COVID-19 pandemic, even in low-volume ECMO centers (43).

Therefore, one of the most pertinent factors in achieving better outcomes requires implementing and maintaining ECMO support without major complications. This complex management can only be achieved with a multidisciplinary team integrated into care. The rigorous assessment of oncological prognosis by oncologists, adequate treatment of pneumonia by pulmonologists, management of hemorrhagic and thrombotic complications by hematologists, high-quality intensive care by critical care physicians and nurses, and safe ECMO initiation and management by cardiac surgeons and perfusionists are necessary. Furthermore, it would be recommended that, given the severity and complexity of these cases, a team specializing in palliative care should be close to the patients and their families, offering psychological and symptom management support for comprehensive and humanized care.

## 6 Conclusion

Given the uncertainty regarding the value of ECMO support in this patient population, the decision to utilize ECMO should involve a multidisciplinary team. Important factors, such as organic dysfunctions, thrombocytopenia or neutropenia, and mainly oncological disease status, can be indicators of poor prognosis and, thus, should be accounted for in the decision regarding ECMO in patients with cancer. Functional status can be a valuable parameter for prospective studies. Furthermore, considering the methodology of the studies evaluated, the oncologic patient prognosis on ECMO requires further investigation. Studies with larger sample sizes and cohorts are needed to create well-defined guidelines to guarantee that intensivists and hemato-oncologists may better approach the management of cancer patients with a possible ECMO indication.
